# Does regular antenatal exercise promote exclusive breastfeeding during the first 3 months of life? Secondary analyses of a randomized controlled trial

**DOI:** 10.18332/ejm/167807

**Published:** 2023-08-25

**Authors:** Eva Marie E. Flaathen, Hege H. Johannessen, Julie Bakke, Cecilie Holm, Siv Mørkved, Kjell Å. Salvesen, Signe N. Stafne

**Affiliations:** 1 Department of Nursing and Health Promotion, Faculty of Health Sciences, Oslo Metropolitan University, Oslo, Norway; 2 Faculty of Health, Welfare and Organisation, Østfold University College, Halden, Norway; 3 Department of Physical Medicine and Rehabilitation, Østfold Hospital Trust, Grålum, Norway; 4 Department of Public Health and Nursing, Norwegian University of Science and Technology, Trondheim, Norway; 5 Department of Rehabilitation, St. Olavs Hospital, Trondheim University Hospital, Trondheim, Norway; 6 Department of Obstetrics and Gynecology, St. Olavs Hospital, Trondheim University Hospital, Trondheim, Norway; 7 Department of Clinical and Molecular Medicine, Norwegian University of Science and Technology, Trondheim, Norway

**Keywords:** risk factors, physical activity, postpartum, health outcomes, exclusive breastfeeding, antenatal exercise

## Abstract

**INTRODUCTION:**

Exclusive breastfeeding (EBF) and antenatal exercise are independently associated with positive short- and long-term health effects for women and their children. The aims of the study were to investigate whether antenatal exercise promotes EBF three months postpartum and further to explore factors associated with EBF at three months postpartum.

**METHODS:**

This study was a follow-up of a Norwegian two-center randomized controlled trial to assess the effect of an antenatal exercise protocol. The recruited pregnant women were randomized to either a 12-week standardized antenatal exercise program with one weekly group training led by a physiotherapist and two weekly home training sessions or standard antenatal care. Women reported breastfeeding status in a questionnaire at three months postpartum.

**RESULTS:**

Of the 726 women, 88% were EBF at three months postpartum. There was no significant difference in EBF rates between the intervention group (87%) and the control group (89%). EBF was positively associated with maternal education (AOR=3.4; 95% CI: 1.7–6.7) and EBF at discharge from the hospital (AOR=22.2; 95% CI: 10–49). Admission to neonatal intensive care unit was identified as a significant barrier to EBF (AOR=0.2; 95% CI: 0.1–0.4). Significantly more women in the non-EBF group had sought professional help compared to women in the EBF group (p≤0.001).

**CONCLUSIONS:**

Regular physical exercise during pregnancy did not influence the exclusive breastfeeding rates at three months postpartum. Considering the health effects of exclusive breastfeeding and antenatal physical exercise, studies with follow-up periods beyond three months postpartum are warranted.

## INTRODUCTION

Exclusive breastfeeding (EBF) means that the infant receives only breast milk without any additional food or drink, except for oral rehydration solutions, drops or syrup preparations, vitamins, minerals, or medicines^1^. WHO recommends women to exclusively breastfeed throughout the first six months postpartum and, after the introduction of solid food at six months, to continue supplementation of breastmilk for two years or more^1,2^, as breast milk contains numerous immunological components that ensure optimal growth, development, and infant health^1,3^.

The WHO recommendations for EBF are based on a robust body of literature supporting improved short- and long-term physical, mental and obstetric health outcomes for mothers and infants in high-, middle-, and low-income countries^1,3-7^.

Despite its established benefits, EBF is not a norm globally. In low- and middle-income countries, only 37% of children younger than six months are exclusively breastfed^3^. In high-income countries, the prevalence of EBF ranges from <10% to approximately 70% in children aged 3–4 months^8^. Although the EBF initiation prevalence rates in Scandinavian countries are among the highest (>98%), the EBF continuation rates decline to 39–68% at four months of age^8-10^. The suggested reasons for the low EBF continuation rates are multifactorial and may be due to cultural, emotional, medical, psychological, or social factors^3,9-15^.

Exercise is defined as physical activity that is planned, structured, and repetitive, aiming to improve or maintain physical fitness^16^. The general adult population, including pregnant women with uncomplicated pregnancies, are encouraged to engage in regular moderate-intensity exercise and physical activity for at least 150 minutes per week^16,17^. However, despite this recommendation, and the well-documented effects of exercise for both mother and offspring^16,18-19^, the proportion of pregnant women who achieve the recommended level of physical activity per week is lower than in non-pregnant women and declines as the pregnancy progresses^16,20,21^.

Although the literature offers indisputable evidence of the independent health advantages of both antenatal exercise^16^ and EBF^1,3,22^, few studies have investigated the association between antenatal exercise and breastfeeding^23,24^. The main aim of this study was to investigate whether an antenatal exercise intervention promotes EBF at three months postpartum compared to standard antenatal care. The secondary aims were to assess the prevalence and potential factors associated with EBF at three months postpartum.

## METHODS

### Study design

This study was a follow-up of the Training in Pregnancy (TRIP) trial, a two-center randomized controlled trial (RCT) conducted in Norway between 2007 and 2009 to evaluate the effect of a 12-week exercise program^25^.

### Sample, setting, and procedure

Pregnant women who were booked for routine ultrasonography at St. Olavs Hospital and Stavanger University Hospital in gestational weeks 18–22 were invited to participate. The inclusion criteria included healthy pregnant women aged ≥18 years with a live singleton fetus. The exclusion criteria were high-risk pregnancies and women who resided more than 30-minutes travel by car from the two hospitals.

Concealed randomization in blocks of 30 was performed using a web-based computerized procedure at the Unit for Applied Clinical Research, Norwegian University of Technology and Science. Women randomized to the exercise program started the intervention within 1–2 weeks after inclusion. The intervention group received a 12-week standardized exercise program, including one weekly physiotherapy-led group training and two weekly home training sessions. The group training session included 30–35 minutes of moderate-intensity aerobic exercise (no running or jumping), 20–25 minutes of strength training, and 5–10 minutes of light stretching and relaxation. In addition, the women were advised to perform a home training program at least twice per week, including 30 minutes of endurance training and 15 minutes of strength training^25^. Intervention group women were encouraged to exercise three days weekly at moderate to high intensity (according to study protocol). Control group women received the standard antenatal care from their midwives or general practitioners, including information about healthy lifestyles, including physical activity^17^. Standard antenatal care is free of charge and a part of the Norwegian public health system. Follow-up was done in gestational weeks 32–36. Both intervention and control group women completed a questionnaire to assess the level of exercise. Women reported on frequency, intensity, and duration of exercise. The proportion of women reporting exercising at moderate to high intensity, minimum of 30 minutes, for three days or more weekly were compared between groups. Data regarding EBF status were collected at three months postpartum during the women’s visit at the outpatient clinic of the two hospitals.

### Ethical considerations

The TRIP trial followed the Declaration of Helsinki. After receiving written and oral information, the women signed the informed consent form. The women did not receive any financial compensation for participating in the study. The study was approved by the Regional Committee for Medical and Health Research Ethics.

### Outcome variable

The primary outcome of this study was the number of women who reported EBF at three months postpartum. Breastfeeding frequency at discharge to home from the hospital was reported retrospectively. The response options were ‘all meals’, ‘three or four meals/day’, ‘two meals/day’, ‘less than two meals/day’, and ‘no breastfeeding’. The women who reported breastfeeding for all meals at each time point were categorized as EBF, and those who reported breastfeeding less frequently were categorized as non-EBF.

### Background variables

Sociodemographic data such as age, body mass index (BMI), education level, work status and marital status were self-reported and obtained at pregnancy weeks 18–22 (inclusion). Information on obstetrical and neonatal exposure variables such as parity, mode of delivery, birth weight, and need for neonatal intensive care were collected from the women’s medical records. Birthweight was categorized as: <2500, 2500–4499, and ≥4500 g.

### Statistical analyses

Descriptive statistics are presented as mean with standard deviation (SD) and range for continuous variables, and as frequencies and percentages for categorical variables. Differences in sociodemographic, socioeconomic, obstetric, and breastfeeding characteristics, including EBF, were assessed using the chi-squared test for categorical variables and the independent-sample t-test or Mann-Whitney U test for continuous variables. All women were included in the analysis according to the intention-to-treat principle.

To explore potential factors associated with EBF at three months postpartum, univariable and multivariable logistic regression analyses were performed with EBF status as the dependent variable and selected obstetric and neonatal background parameters as independent variables. Variables with p<0.2 in the univariable analyses were included in the multivariable logistic regression model. Multivariable logistic regression analyses were performed using stepwise backward selection to evaluate the independent strengths of the associations between EBF status and the selected independent variables. The variable with the highest p-value was excluded from the model at each step until all variables were statistically significant. Effect estimates are presented as adjusted odds ratios (AORs) and 95% confidence intervals (CIs). None of the variables included in the final multivariable logistic regression model showed a high correlation (variance influence factor <2.0). A significance level of 5% was used throughout. IBM SPSS Statistics version 27 for Windows was used in all the statistical analyses.

## RESULTS

**Figure 1:**
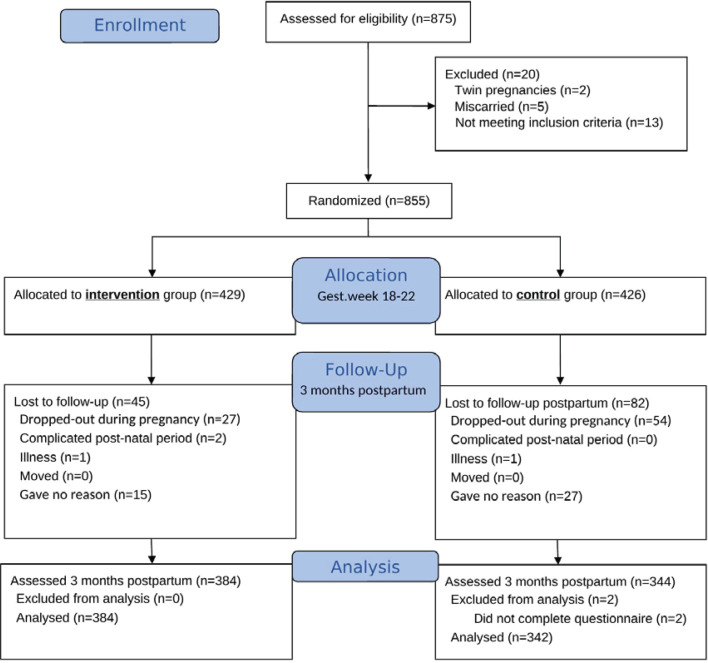
CONSORT 2010 flow diagram

In the original randomized controlled trial, 429 and 426 women were randomly allocated to the intervention and control groups, respectively^25^. At three months postpartum, 129 women did not respond, and data from 384 women in the intervention group and 342 women in the control group were included in the analyses (Figure 1).

Women in the intervention and control groups had similar baseline characteristics (Table 1). The mean age was 30.6 years, and 424 women (58%) were nulliparous. Most women were married or living with their partners and employed (Table 1). In gestational weeks 32–36, 56% of women in the intervention group reported exercise training according to the study protocol (3 times per week) compared to 11% in the control group (p<0.001).

**Table 1:** Participant characteristics according to randomization group and breastfeeding status among women in the Training in Pregnancy trial, Norway 2007–2009 (N=726)

*Characteristics*	*Allocation in original RCT (baseline data)*			*Breastfeeding status at 3 months postpartum*		
	*Control group (n=342)*	*Intervention group (n=384)*		*Exclusive breastfeeding (n=638)*	*Non-exclusive breastfeeding (n=88)*	
	*n (%)*	*n (%)*	*p*	*n (%)*	*n (%)*	*p*
**Age** (years), mean (SD), [range]	30.6 (4.2) [4–20]	30.6 (4.3) [20–46]	0.574*	30.6 (4.1) [20–46]	30.6 (4.9) [20–44]	0.996*
**BMI** (kg/m^2^), mean (SD), [range]	23.1 (3.3) [3–17]	23.0 (2.9) [18–36]	0.210*	22.9 (2.9) [17–36]	23.9 (4.1) [17–38]	0.003*
**Randomisation**						0.310**
Control group	-	-		305 (48)	37 (42)	
Intervention group	-	-		333 (52)	51 (58)	
**Parity**			0.973**			0.434**
Primiparous	200 (58)	224 (58)		376 (59)	48 (55)	
Multiparous	142 (42)	160 (42)		262 (41)	40 (45)	
**Education level** (years)			0.075**			<0.001**
<13	37 (11)	28 (7.2)		46 (7.2)	19 (22)	
≥13	305 (89)	356 (93)		592 (93)	69 (78)	
**Employed**	326 (95)	360 (94)	0.358**	602 (95)	84 (96)	0.771**
Missing	1 (0.3)	0		1 (0.3)	0	
**Married/live-in-partner**	338 (99)	374 (97)	0.072**	625 (98)	85 (97)	0.346**
**Mode of delivery**			0.483***			0.022***
Normal vaginal	263 (77)	291 (75)		495 (78)	57 (65)	
Instrumental	41 (12)	40 (10)		84 (13)	11 (13)	
Cesarean section	37 (11)	55 (14)		57 (9.0)	20 (23)	
Missing	1 (0.3)	1 (0.3)				
**Birthweight** (g)			0.460***			0.180***
<2500	10 (2.9)	13 (3.4)		16 (2.5)	7 (8.0)	
2500–4499	320 (94)	367 (95)		604 (95)	80 (91)	
>4500	12 (3.5)	7 (1.8)		18 (2.8)	1 (1.1)	
Missing	0	1 (0.3)				
**Transferred NICU**	15 (4.4)	12 (3.1)	0.365**	14 (2.2)	13 (14)	<0.001**
Missing	6 (1.8)	5 (1.3)		9 (1.4)	1 (1.1)	
**EBF on discharge home from hospital**	325 (94)	364 (94)	0.749**	627 (98.3)	62 (71)	<0.001**
Missing	1 (0.3)	3 (0.8)				
**Self-reporting exercising 3 days per week at gestational 32–36 weeks**	36 (11)	214 (56)	<0.001**	220 (35)	30 (34)	0.782**
Missing	10 (2.9)	6 (1.6)		10 (1.6)	6 (6.8)	

* Independent sample’s t-test.

* *Chi-squared test.

*** Mann Whitney U test.

BMI: body mass index. NICU: neonatal intensive care unit. EBF: exclusive breast feeding. Exercise training according to study protocol: training at moderate/high intensity three times or more per week.

A total of 638 women (88%) were EBF at three months postpartum, and there were no significant differences between randomization groups (p=0.310) (Table 1). The reported reasons for not EBF were similar in both groups (Table 2).

**Table 2:** Breastfeeding frequency at 3 months, reasons for not breastfeeding and seeking professional help with breastfeeding according to randomization group and breastfeeding status at 3 months in the Training in Pregnancy trial, Norway 2007–2009 (N=726)

	*Allocation in original RCT (baseline data)*			*Breastfeeding status at 3 months postpartum*		
	*Control group (n=342)*	*Intervention group (n=384)*		*Exclusive breastfeeding (n=638)*	*Non-exclusive breastfeeding (n=88)*	
	*n (%)*	*n (%)*	*p*	*n (%)*	*n (%)*	*p*
**Breastfeeding at 3 months**			0.342*			-
Exclusive breastfeeding	305 (89)	333 (87)		638 (100)	-	
3–4 times/day	12 (3.59)	21 (5.4)		-	33 (38)	
2 times/day	6 (1.7)	7 (1.8)		-	13 (15)	
<2 times/day	1 (0.3)	3 (0.89		-	4 (4)	
Not breastfeeding	18 (5.2)	20 (5.2)		-	38 (43)	
**Reasons for not breastfeeding**	(n=41)	(n=56)	0.811**		(n=86)	-
Not enough milk	21 (6.1)	31 (8.0)		-	45 (51)	
Sore nipples	3 (0.9)	8 (2.1)		-	11 (13)	
Other	17 (4.9)	17 (4.4)		-	30 (34)	
Missing	-	-		-	2 (2.3)	
**Have you sought professional help?**			0.940*			<0.001*
Never	111 (33)	143 (37)		143 (37)	16 (18)	
Once	58 (17)	60 (16)		104 (16)	14 (16)	
2–4 times	47 (14)	58 (15)		76 (12)	29 (33)	
>4 times	17 (5.0)	28 (7.2)		24 (3.8)	21 (24)	
Missing	109 (32)	98 (25)		196 (31)	8 (9)	

* Mann Whitney U test.

** Chi-squared test.

The analyses that compared baseline background characteristics of the responders and women who were lost to follow-up at three months postpartum showed significant differences between the groups regarding age, BMI, parity, employment status and randomization status (Supplementary file Table 1). The differences in mean age and BMI were less than one year (30.6 vs 29.7), and only 0.8 (23.0 vs 23.8), respectively. More women who were unemployed and had less than 13 years of education were lost to follow-up. Furthermore, fewer women in the control group (47%) responded at three months postpartum compared to 53% in the intervention group.

Compared to non-EBF women, significantly more EBF women had a higher educational level, and nearly all women (98% vs 71%, p≤0.001) were EBF at discharge to home after delivery. The non-EBF women had higher pre-pregnancy BMI values (23.9 vs 22.9 kg/m^2^), and 14% of them had infants admitted to the neonatal intensive care unit (NICU) after delivery compared to only 2% of the EBF women (Table 1). Among EBF women, fewer than one in ten were delivered by cesarean section, and 78% had a normal vaginal birth. In comparison, nearly one in four non-EBF women delivered by cesarean section, and 65% had a normal vaginal birth (Table 1).

One in three women in the non-EBF group was breastfeeding three to four meals daily, whereas almost half were not breastfeeding at three months postpartum (Table 2). The most common reason for not breastfeeding was that women did not have enough breast milk (Table 2). Significantly more women in the non-EBF group reported having sought professional help more than once (p≤ 0.001) (Table 2).

In the univariate logistic regression analysis, cesarean section, age, pre-pregnancy BMI, and birthweight <2500 g were significantly associated with reduced odds of EBF at three months. However, these associations did not remain significant in the multivariable model. In the multivariable model, women with a higher educational level were more than three times as likely to be EBF at three months postpartum (p<0.01). Furthermore, the women whose infants were transferred to the NICU postpartum were less likely to be EBF at three months than those with healthy infants (AOR=0.2; 95% CI: 0.1–0.2). EBF at hospital discharge after delivery was significantly associated with EBF at three months postpartum (AOR=22; 95% CI: 10–49) (Table 3).

**Table 3:** Factors associated with exclusive breastfeeding (EBF) at three months postpartum among women in the Training in Pregnancy trial, Norway 2007-2009 (N=715)

	*Univariable logistic regression*	*Multivariable logistic regression*
	*OR (95% CI)*	*AOR (95% CI)*
**Age** (years)	**1.0 (0.9–1.1)**	-
**Prepregnancy BMI**	**0.9 (0.8–0.9)**	
**Control vs intervention group**	0.8 (0.5–1.2)	
**Primipara vs multipara**	0.8 (0.5–1.3)	
**<13 years vs ≥13 years education**	**3.5 (2.0–6.3)**	**3.4 (1.7–6.7)**
**Unemployed vs employed**	0.8 (0.3–2.4)	
**Married/live-in-partner vs single**	1.8 (0.5–6.5)	
**Mode of delivery**		
Normal vaginal (Ref.)	1	
Instrumental	**0.3 (0.2–0.6)**	-
Cesarean section	0.9 (0.4–1.8)	-
**Birthweight** (g)		
<2500	**0.3 (0.1–0.8)**	-
2500–4499 (Ref.)	1	
>4500	2.4 (0.3–18.0)	-
**Transferred NICU**	**0.1 (0.1–0.3)**	**0.2 (0.1–0.4)**
**EBF on discharge home from hospital**	**23.0 (11–44)**	**22.2 (10–49)**

AOR: adjusted odds ratio. BMI: body mass index (kg/m^2^). NICU: neonatal intensive care unit. EBF: exclusively breastfeeding.

## DISCUSSION

Our main finding is that the overall prevalence of EBF was high. However, there were no significant differences in EBF rates at three months postpartum between the randomization groups. Significantly more non-EBF women at three months postpartum had sought professional help compared to women who were EBF. In the multivariable analyses, EBF was significantly associated with a higher maternal educational level and EBF at discharge to home from the hospital, whereas admission to the NICU was negatively associated with EBF three months postpartum.

To the best of our knowledge, this is the first trial to investigate the effect of an antenatal exercise program on EBF rates at three months postpartum compared to standard antenatal care. Previous trials focusing on postpartum exercise and breastfeeding outcomes have found no adverse effects on breast milk volume and composition^26^ or breastfeeding duration^23^. Due to the positive short- and long-term health effects^1,3,16,18,19^, further research on the effects of exercise during pregnancy and EBF rates with follow-up periods >3 months may be warranted.

In our study, EBF at three months was reported by 88%. This prevalence is higher than reported by national surveys^9,10,12^ and studies from high-, middle-, and low-income countries^3,8^. In a review, the global prevalence of EBF at three to four months varies from approximately 70% in Norway to <10% in the United Kingdom^8^. In a meta-analysis investigating the prevalence of EBF in children aged <2 years, information about EBF at 0–5 months in high-income countries was unavailable, whereas EBF ranged from 30% to 45% in low- and middle-income countries^3^. However, due to the differences in data collection, breastfeeding definitions, and participant selection, comparing studies is difficult.

One interesting finding was that significantly more women who were not EBF at three months postpartum had sought professional help with breastfeeding than women who were EBF at three months. Although the EBF initiation prevalence rate in Norway is among the highest in high-income countries, the continuation rate declines significantly at three to four months of age^8-10^. Due to the established health benefits of EBF^3-7^, the current EBF durations and exclusivity rates are not at ideal levels^1,2^. To achieve substantial improvements in EBF rates, efficacious intervention strategies must be identified, and the development, implementation, and examination of interventions from the prenatal to the postpartum period are warranted to improve breastfeeding outcomes at six months postpartum^11,22^. The interventions should include education about the health benefits for mothers and infants, lactation, breastfeeding techniques, and social and psychological support^11^.

The present study demonstrates a significant association between EBF at discharge to home from the hospital and three months postpartum. The efficacy of the WHO’s Baby-friendly Hospital Initiative is well documented and recommends at least one hour of skin-to-skin contact between mother and infant during the first hour after delivery to improve breastfeeding initiation, maintenance, and duration^3,11,13^. Hospitals in Norway follow the Baby-friendly Hospital Initiative to support EBF. Studies have found that the separation of mothers and infants soon after birth is a crucial risk factor preventing skin-to-skin contact, and thus initiation of breastfeeding and EBF in the short- and long-term^12-14^. In our study, transfer to the NICU was negatively associated with EBF at three months postpartum. Delayed skin-to-skin contact prevents oxytocin excretion and breast milk production, increasing the likelihood of the infant receiving formula before the first breastfeeding^14^. Results from the Norwegian Mother and Child Cohort Study showed that supplementary feeding with formula, water, or sugar water during the first week of life increased the risk of EBF cessation during not only the first month but also through the second and third months of an infant’s life^12^. If a term infant is transferred to the NICU and skin-to-skin contact is delayed, clinical practices supporting the mother to provide breast milk for her infant must be identified and promoted to enable EBF.

We found a significant association between EBF at three months postpartum and maternal education level, consistent with previous findings. A review of studies from high-income countries showed that women with higher income and education level tended to breastfeed more than those with low income and fewer years of formal education^3^. A Norwegian population-based prospective cohort study among 1490 women showed maternal education was associated with EBF up to four months postpartum^10^. This concurs with a Danish cohort study among 471 women investigating sociodemographic and psychosocial factors related to the length of EBF^27^. To help mothers who want to EBF, health professionals must identify women at risk of early EBF cessation throughout the pregnancy and postpartum periods and develop strategies to promote EBF.

Maternal overweight and obesity are associated with adverse health consequences for both mothers and infants^15,21^. Moreover, breastfeeding initiation and duration is associated with a more rapid return to pre-pregnancy weight and prevention of obesity and overweight later in life^15^. In the present study, the association between EBF and BMI was not statistically significant in the multivariable analyses. However, the pre-pregnancy BMI in our study’s healthy, low-risk women was lower than the average BMI in the general population of Norwegian women who gave birth in 2019 (BMI: 24.6 ± 4.9 kg/m^2^; Norwegian Birth Registry 2019). Anatomical, sociocultural, medical, and psychosocial factors are associated with lower EBF rates in overweight and obese women, and maternal obesity (BMI ≥25 kg/m^2^) negatively affects breastfeeding intention, initiation, intensity, and duration^15,21^. Thus, an increased focus on breastfeeding in the prenatal and postpartum periods among overweight and obese women is vital.

### Strengths and limitations

The strengths of this study are the prospective data collection, the high number of participants, and the exploration of the effect of an antenatal exercise program on EBF rates at three months postpartum. The main limitation is the secondary analysis that was not pre-specified in the study protocol. Only 56% of intervention group women adhered to the study protocol, and conclusions regarding the effect of antenatal exercise training cannot be drawn. Further, the included women were mainly healthy, white European, normal-weight, and highly educated, and this may reduce the external validity. Thus, our results should be interpreted with caution in groups with diverse ethnic and cultural backgrounds and low socioeconomic status.

Lost to follow-up analyses showed statistically significant differences regarding age, BMI, parity, employment status, and randomization status. The differences were small, and these minor differences are unlikely to have any clinical meaningful influence on the results. We also found that more women who were unemployed and had less than 13 years of education were lost to follow-up. This is in concurrence with previous research^28^. Fewer women in the control group responded at three months postpartum than women in the intervention group. It may be that having participated in active training during pregnancy may have motivated these women more than those who received standard antenatal care. This may have influenced our results at three months. However, randomization was not significantly associated with exclusive breastfeeding at three months in our univariable logistic regression analyses and was thus not included in the multivariable analyses (Table 3).

Data on EBF at discharge to home from the hospital were collected retrospectively and might have been affected by recall bias. However, studies show that mothers provide accurate estimates of breastfeeding initiation and duration within a recall period of up to three years^29^. The women in our trial reported EBF in one question, thus, we cannot fully conclude that their answers were based on the WHO’s definition of EBF.

## CONCLUSIONS

The moderate-intensity exercise program during pregnancy did not affect the EBF rates at three months postpartum. However, the overall rate of EBF was high in our population, and significantly more non-EBF women had sought professional breastfeeding help than EBF women. A higher education level and EBF at discharge to home from the hospital increased the odds, whereas transfer to the NICU was identified as an obstacle to EBF at three months postpartum. Considering the independent short- and long-term health benefits of antenatal physical exercise and EBF for mothers and infants, studies with follow-up periods beyond three months postpartum in culturally diverse, sociodemographic, and socioeconomic populations are warranted.

## Data Availability

The data supporting this research are available from the authors on reasonable request.

## References

[1] World Health Organization (2017). Exclusive breastfeeding for optimal growth, development and health of infants.

[2] Kramer MS, Kakuma R (2012). Optimal duration of exclusive breastfeeding. Cochrane Database Syst Rev.

[3] Victora CG, Bahl R, Barros AJ (2016). Breastfeeding in the 21st century: epidemiology, mechanisms, and lifelong effect. Lancet.

[4] Duijts L, Ramadhani MK, Moll HA (2009). Breastfeeding protects against infectious diseases during infancy in industrialized countries. A systematic review. Matern Child Nutr.

[5] Hauck FR, Thompson JM, Tanabe KO, Moon RY, Vennemann MM (2011). Breastfeeding and reduced risk of sudden infant death syndrome: a meta-analysis. Pediatrics.

[6] Saxton A, Fahy K, Rolfe M, Skinner V, Hastie C (2015). Does skin-to-skin contact and breast feeding at birth affect the rate of primary postpartum haemorrhage: Results of a cohort study. Midwifery.

[7] Stuebe AM, Schwarz EB (2010). The risks and benefits of infant feeding practices for women and their children. J Perinatol.

[8] Ibanez G, Martin N, Denantes M, Saurel-Cubizolles MJ, Ringa V, Magnier AM (2012). Prevalence of breastfeeding in industrialized countries. Rev Epidemiol Sante Publique.

[9] Myhre JB, Andersen LF, Kristiansen AL (2020). Spedkost 3. Landsomfattende undersøkelse av kostholdet blant spedbarn i Norge, 6 måneder. Rapport 2020.

[10] Kristiansen AL, Lande B, Øverby NC, Andersen LF (2010). Factors associated with exclusive breast-feeding and breast-feeding in Norway. Public Health Nutr.

[11] Cordell A, Elverson C (2021). Interventions to Improve Breastfeeding Outcomes from Six Weeks to Six Months: A Systematic Review. West J Nurs Res.

[12] Häggkvist AP, Brantsæter AL, Grjibovski AM, Helsing E, Meltzer HM, Haugen M (2010). Prevalence of breast-feeding in the Norwegian Mother and Child Cohort Study and health service-related correlates of cessation of full breast-feeding. Public Health Nutr.

[13] McInnes RJ, Chambers J (2008). Infants admitted to neonatal units – interventions to improve breastfeeding outcomes: a systematic review 1990–2007. Matern Child Nutr.

[14] Moore ER, Bergman N, Anderson GC, Medley N (2016). Early skin-to-skin contact for mothers and their healthy newborn infants. Cochrane Database Syst Rev.

[15] Turcksin R, Bel S, Galjaard S, Devlieger R (2014). Maternal obesity and breastfeeding intention, initiation, intensity and duration: a systematic review. Matern Child Nutr.

[16] (2020). Physical Activity and Exercise During Pregnancy and the Postpartum Period: ACOG Committee Opinion, Number 804. Obstet Gynecol.

[17] Svangerskapsomsorgen: Nasjonal faglig retningslinje. Website in Norwegian.

[18] Coll CVN, Domingues MR, Stein A (2019). Efficacy of Regular Exercise During Pregnancy on the Prevention of Postpartum Depression: The PAMELA Randomized Clinical Trial. JAMA Netw Open.

[19] Sobierajski FM, Purdy GM, Usselman CW (2018). Maternal Physical Activity Is Associated With Improved Blood Pressure Regulation During Late Pregnancy. Can J Cardiol.

[20] Gaston A, Cramp A (2011). Exercise during pregnancy: a review of patterns and determinants. J Sci Med Sport.

[21] Guelinckx I, Devlieger R, Beckers K, Vansant G (2008). Maternal obesity: pregnancy complications, gestational weight gain and nutrition. Obes Rev.

[22] Rollins NC, Bhandari N, Hajeebhoy N (2016). Why invest, and what it will take to improve breastfeeding practices?. Lancet.

[23] Nguyen PTH, Binns CW, Nguyen CL (2019). Physical Activity During Pregnancy is Associated with Improved Breastfeeding Outcomes: A Prospective Cohort Study. Int J Environ Res Public Health.

[24] Tucker EA, Fouts HN (2017). Connections Between Prenatal Physical Activity and Breastfeeding Decisions. Qual Health Res.

[25] Stafne SN, Salvesen KÅ, Romundstad PR, Eggebø TM, Carlsen SM, Mørkved S (2012). Regular exercise during pregnancy to prevent gestational diabetes: a randomized controlled trial. Obstet Gynecol.

[26] Dewey KG, Lovelady CA, Nommsen-Rivers LA, McCrory MA, Lönnerdal B (1994). A randomized study of the effects of aerobic exercise by lactating women on breast-milk volume and composition. N Engl J Med.

[27] Kronborg H, Vaeth M (2004). The influence of psychosocial factors on the duration of breastfeeding. Scand J Public Health.

[28] Nohr EA, Liew Z (2018). How to investigate and adjust for selection bias in cohort studies. Acta Obstet Gynecol Scand.

[29] Li R, Scanlon KS, Serdula MK (2005). The validity and reliability of maternal recall of breastfeeding practice. Nutr Rev.

