# Supplementary material for: Does regular antenatal exercise promote exclusive breastfeeding during the first 3 months of life? Secondary analyses of a randomized controlled trial
Source: Eur J Midwifery. 2023 Aug 25;7:20. doi: 10.18332/ejm/167807 (PMC10450771; doi:10.18332/ejm/167807)
Supplement: Supplementary file 1 [file EJM-7-20-s1.pdf]

Supplementary Table 1. Characteristics of responders and non-responders at follow-up three months postpartum among women tin the Training in Pregnancy trial, Norway 2007-2009 (N=732)

	**Responders (n=732)**	**Non-responders (n=129)**	p-value
**Age** years, mean (SD)[range]	30.6 (4.2) [20,46]	29.7 (4.9) [19,42]	0.011*
**BMI** kg/m^2 mean (SD)[range]	23.0 (3.1) [17.1,38.4]	23.8 (3.7) [17.3,36.7]	0.013*
**Randomisation**			<0.001**
Control group	342 (47%)	84 (65%)	
Intervention group	384 (53%)	45 (35%)	
**Parity**			0.029**
Primiparous	424 (58%)	62 (48%)	
Multiparous	302 (42%)	67 (52%)	
**Education**			<0.001**
≤ 13 years education	65 (9%)	30 (23%)	
≥13 years education	661 (91%)	99 (77%)	
**Employed**	686 (95%)	112 (87%)	<0.001**
**Married/live-in-partner**	710 (98%)	124 (96%)	0.211**

Results are presented as n(%) unless stated otherwise. *=Independent sample's t-test; **=chi-squared test

© 2023 Flaathen E. M. E. et al.
